# Gut microbiota metabolites and risk of major adverse cardiovascular events and death: A systematic review and meta-analysis

**DOI:** 10.1097/MD.0000000000037825

**Published:** 2024-05-31

**Authors:** Qaisar Ali Khan, Muhammad Asad, Abdul Hannan Ali, Ameer Mustafa Farrukh, Usama Naseem, Bader Semakieh, Yaxel Levin Carrion, Muhammad Afzal

**Affiliations:** a Khyber Teaching Hospital, MTI KTH, Peshawar, Pakistan; b Lady Reading Hospital Peshawar, Pakistan; c Khyber Medical College, Peshawar, Pakistan; d University of Galway School of Medicine, Galway, Ireland; e Combined Military Hospital, CMH, Peshawar, Pakistan; f Arkansas College of Osteopathic Medicine, Fort Smith, AR; g Rutgers New Jersey Medical School, Newark, NJ; h St. George’s University School of Medicine, True Blue, Grenada.

**Keywords:** major adverse cardiovascular events, mortality, risk, trimethylamine N-oxide

## Abstract

**Background:**

Gut microbial metabolites such as trimethylamine N-oxide (TMAO) and its precursors, namely betaine, L-carnitine, and choline, have been implicated as risk factors for cardiovascular events and mortality development. Therefore, we aim to perform a systematic review and meta-analysis to assess the validity of these associations.

**Methods:**

MEDLINE and Scopus were queried from their inception to August 2023 to identify studies that quantified estimates of the associations of TMAO with the development of major adverse cardiovascular events (MACE) or death. A random-effects meta-analysis was conducted to pool unadjusted or multivariable-adjusted hazard ratios (HR) and their 95% confidence intervals. The primary endpoint was the risk of MACE and all-cause death.

**Results:**

30 prospective observational studies (n = 48 968) were included in the analysis. Elevated TMAO levels were associated with a significantly greater risk of MACE and all-cause death compared to low TMAO levels (HR: 1.41, 95% CI 1.2–1.54, *P* < .00001, *I*^2^ = 43%) and (HR: 1.55, 95% CI 1.37–1.75, *P* < .00001, *I*^2^ = 46%), respectively. Furthermore, high levels of either L-carnitine or choline were found to significantly increase the risk of MACE. However, no significant difference was seen in MACE in either high or low levels of betaine.

**Conclusion:**

Elevated concentrations of TMAO were associated with increased risks of MACE and all-cause mortality. High levels of L-carnitine/choline were also significantly associated with an increased risk of MACE. However, no significant difference was found between high or low levels of betaine for the outcome of MACE.

## 1. Introduction

There is accumulating evidence that gut microbiota plays a crucial role in regulating host cardio-metabolic functioning by modulating the concentrations of bioactive metabolites.^[1,2]^ One such metabolite, trimethylamine N-oxide (TMAO), mainly derived from choline, has recently been shown to be associated with cardiovascular diseases and events.^[3]^ Choline (commonly obtained from the consumption of red meat, poultry, and eggs) is metabolized by the gut microbiota to produce trimethylamine (TMA), which is then converted to TMAO via the hepatic enzyme flavin monooxygenase.^[4]^ A recent study conducted by Zhang et al^[5]^ revealed that being in the higher TMAO category significantly increased the odds of stroke.

Several studies have evaluated an association between elevated blood concentrations of TMAO and its precursors (betaine, L-carnitine, and choline) with major adverse cardiovascular events (MACE) and all-cause death.^[6–8]^ Higher plasma TMAO levels have been associated with a significant 3.0-fold increase in the risk of MACE and a 3.6-fold increase in the risk of mortality in patients with type 2 diabetes mellitus.^[9,10]^ However, inconsistencies owing to different patient populations also exist.^[11–14]^

A previous meta-analysis by Heianza et al^[15]^ analyzed 19 prospective studies from 16 publications to evaluate the relationship of TMAO and its precursors with the development of MACE and all-cause mortality. They found elevated concentrations of TMAO to be associated significantly associated with MACE and all-cause mortality. However, several new investigations have been published assessing the impact of TMAO levels on the risk of MACE and all-cause mortality.^[10,16]^ Hence, this systematic review and meta-analysis aim to quantify the interrelation of blood concentrations of TMAO and its precursors, betaine and L-carnitine or choline, with the risk of MACE and all-cause death using prospective observational studies.

## 2. Methods

### 2.1. Data sources and search strategy

This systematic review and meta-analysis followed the PRISMA (Preferred Reporting Items for Systematic Review and Meta-Analysis) guidelines.^[17]^ A PRISMA search strategy utilizing Boolean operators and PICO (Patient, Intervention, Control, and Outcomes) criteria was used to search databases such as MEDLINE, Google Scholar, and Embase from inception till August 2023. The work has been reported in line with AMSTAR (Assessing the methodological quality of systematic reviews) guidelines. Studies reporting the risk of MACE and all-cause death categorized by blood concentrations of TMAO and its precursors were identified. Two independent authors conducted the literature screening, and conflicts were resolved by discussion and consensus with a third author. The following keywords and their MeSH (medical subject headings) terms were used in this literature search: trimethylamine (N-oxide [text] OR TMAO [text]) AND (atherosclerosis [text] OR death [text] OR mortality [text], OR stroke [text], OR heart failure [text], OR coronary [text], OR cardiovascular [text], OR cerebrovascular disorders [text], OR cardiovascular diseases [Mesh]. No restrictions were applied based on language, author names, year of publication, and country or institution. The detailed search strategy has been reported in Table S1, Supplemental Digital Content, http://links.lww.com/MD/M235 of the supplementary material.

### 2.2. Study selection

Once the literature search was completed, the identified articles were exported to the Endnote Reference Library Software (Version X7.5; Clarivate Analytics, Philadelphia, PA). A duplicate filter was applied to ensure that duplicates present in several online databases were removed. The remaining articles were screened thoroughly based on the title and abstract by 2 independent authors to ensure they satisfied the eligibility criteria. All prospective studies satisfying the following criteria were included in our analysis: blood concentrations of TMAO were the exposure of interest, the outcome of interest was MACE or all-cause mortality, and the studies reported Hazard ratios (HRs) with 95% confidence intervals (CIs) for quantitative categories of TMAO levels.

### 2.3. Study outcomes (main and secondary analysis)

The primary endpoint was the effect of TMAO concentrations on the risk of MACE. High TMAO groups were indicated by the highest tertile (10 studies), the top 2 tertiles (1 study), the highest quartile (13 studies), and the highest quintile (4 studies). Some studies stratified results by disease status at baseline or ethnicity, and those were treated as 2 separate data points in the overall analysis. In our analysis, MACE was defined as a composite of myocardial infarction (MI), stroke, heart transplant, heart failure, other ischemic cardiovascular events, or death (either cardiovascular or all-cause). The secondary endpoint included all-cause mortality alone. We also assessed the effect of the concentrations of TMAO precursors (Betaine and choline/L-carnitine) on the risk of MACE. Furthermore, we evaluated the risk of MACE per 1 SD log-transformed increment of TMAO.

### 2.4. Data extraction

Two independent authors conducted data extraction of relevant articles. In each study, the following data was extracted: a) study name and year, c) study duration, d) total number of participants, e) general patient characteristics (mean age, percentage of males), f) mean or median and interquartile ranges (IQR) for TMAO concentrations, g) all outcomes of interest.

### 2.5. Study quality assessment

Two authors independently assessed the quality of all prospective studies using the Newcastle–Ottawa quality scale for non-randomized studies.^[18]^ Any discrepancy was resolved by discussion and consensus.

### 2.6. Statistical analysis

This meta-analysis used Review Manager (RevMan) Version 5.4 Cochrane Collaboration. A random-effects meta-analysis was conducted to pool unadjusted or multivariable-adjusted hazard ratios (HR) and their 95% confidence intervals. A *P* value < .05 was considered statistically significant for all outcomes. The pooled results are presented as forest plots. Heterogeneity was evaluated using Higgin’s *I*^2^ value. A value of *I*^2^ = 25–50% was considered mild, 50–75% moderate, and > 75% severe heterogeneity. Studies with high heterogeneity were subjected to sensitivity analysis to assess the difference in the significance of the outcomes.

### 2.7. Quality assessment and publication bias

Most of the studies included in the analysis obtained a score of 8 or 9 when evaluating the risk of bias, owing to their robust methodology. Detailed quality assessment has been reported in Table S2, Supplemental Digital Content, http://links.lww.com/MD/M236 of the supplementary material. Following the Cochrane guidelines, funnel plots constructed to determine publication bias for the outcome of MACE and all-cause mortality showed significant bias as the studies scattered asymmetrically around the summary effect size (Figure S4, Supplemental Digital Content, http://links.lww.com/MD/M233 and Figure S5, Supplemental Digital Content, http://links.lww.com/MD/M234). Publication bias was not determined for outcomes reported by fewer than 10 studies.

## 3. Results

### 3.1. Study selection and study characteristics

**Figure 1.**
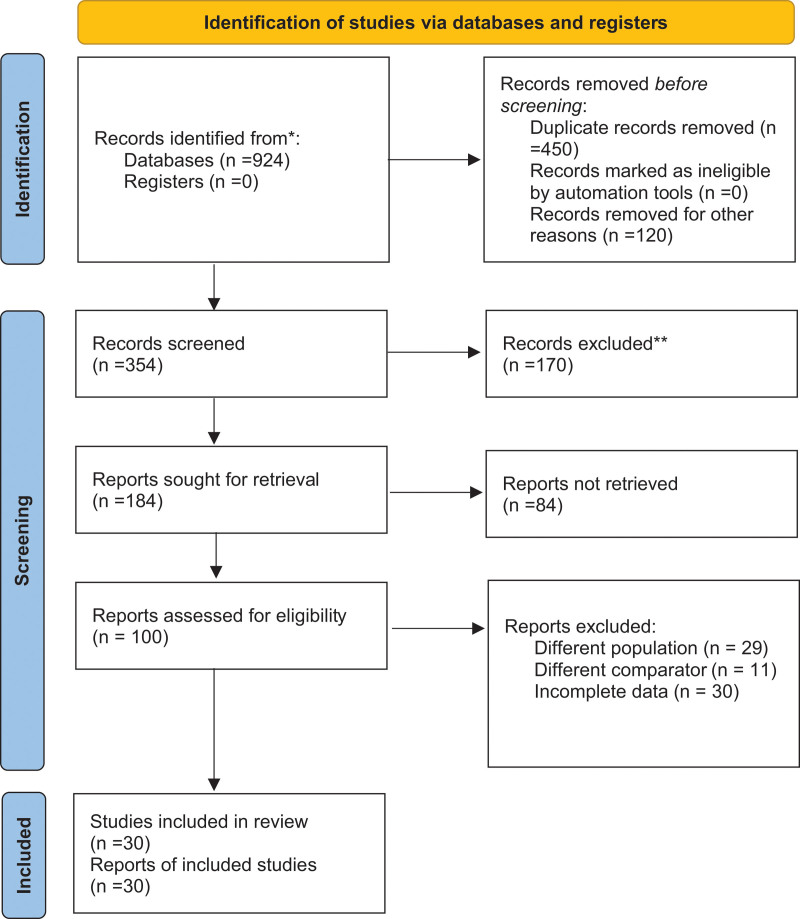
PRISMA flow diagram for new systematic reviews, which included searches of databases and additional records.

A total of 924 studies were retrieved initially from all databases. After excluding and checking for eligibility, 30 studies were included in this meta-analysis. The summary of this procedure is indicated in the PRISMA chart shown in Figure 1. A total of 30 prospective observational studies with a total of 48,968 participants were included in the analysis.^[19–48]^ The mean or median value of TMAO ranged from 1.74 to 103.8 μmol/L across all the studies. Table 1 summarizes the baseline characteristics of all the included studies.

**Table 1:** Baseline characteristics of the included studies.

Study, year	Total participants, N	Mean Age, years	Men, %	Comparison	Patient population	Follow-up, years	TMAO, mean or median; ranges, μmol/L	Outcomes
Tang,^[1]^ (2013)^[19]^	4007	63.0	64.0	Highest quartile vs lowest	Patients undergoing elective coronary angiography	Up to 3 yr	3.7; 2.4–6.2 (IQR)	Death, MI, or stroke
Koeth, (2013)^[21]^	2595	62	70	Above median levels of TMAO vs below median levels of TMAO	Patients undergoing elective cardiac evaluation,	3 yr	NR	MACE (composite of death, MI, stroke, and revascularization)
Lever, (2014)^[20]^	396	68.0	72.7	Highest quintile vs non highest quintile	Participants without diabetes mellitus	4.96 yr (median)	4.8; 3.0–29.1 (IQR)	Cardiovascular disease (CVD) events, all-cause mortality
	79	74.0	73.4	Highest quintile vs non highest quintile	Participants with diabetes mellitus	4.82 yr (median)	7.5; 4.4–12.1 (IQR)	CVD events, all-cause mortality
Tang,^[2]^ (2014)^[22]^	720	66.0	59.0	Highest quartile vs lowest	Patients with stable heart failure (HF) undergoing cardiac evaluation	Up to 5 yr	5.0; 3.0–8.5 (IQR)	All-cause mortality
Wang, (2014)^[23]^	3903	63	64	Above median levels of TMAO vs below median levels of TMAO	Patients undergoing elective diagnostic coronary angiography	3 yr	3.7; 2.4–6.2 (IQR)	MACE (death, MI, stroke)
Kaysen, (2015)^[24]^	235 (whites) 152 (blacks)	61.8	55.3	Highest quartile vs lowest	Comprehensive Dialysis Study	4 yr (median) 2.5 yr (median)	43.0; 28–67 (IQR)	All-cause mortality, CVD death or hospitalization
Troseid, (2015)^[26]^	155	57.0	83.0	Highest tertile vs remaining groups	Patients with stable HF	5.2 yr (median)	9.2 in patients with dilated cardiomyopathy; 12.1 in patients with stable CAD; 1.2–124 (range)	All-cause mortality or heart transplantation
Tang,^[3]^ (2015)^[25]^	521	70.0	48.0	Highest quartile vs lowest	Patients with CKD who underwent elective diagnostic coronary angiography for cardiac evaluation	Up to 5 yr	7.9; 5.2–12.4 (IQR)	All-cause mortality
	3166	62.0	66.0	Highest quartile vs lowest	Patients without CKD who underwent elective diagnostic coronary angiography	Up to 5 yr	3.4; 2.3–5.3 (IQR)	All-cause mortality
Suzuki, (2016)^[27]^	972	78.0	61.0	Highest tertile vs lowest	Patients with acute HF	Up to 1 yr	5.6; 3.4–10.5 (IQR); 0.5–151.5 (range)	In-hospital mortality, All-cause mortality, Death or rehospitalization because of HF
Missailidis, (2016)^[28]^	179	55.0	65.0	Top 2 tertiles vs lowest	Patients with CKD	Up to 5 yr	53.4; 9.3, 170.0 (10th, 90th)	All-cause mortality
Stubbs, (2016)^[29]^	220	69.7	42.7	Highest tertile vs lowest	Patients with CKD, Diabetes Genome Project	Up to 4 yr	6.9; 4.8–10.9 (IQR); 0.63–163.03 (range)	All-cause mortality
Kim, (2016)^[30]^	2529	68.2	62.5	Highest quartile vs lowest	Patients with CKD	3 yr	20.41; 12.82–32.70 (IQR)	Ischemic cardiovascular events
Senthong,^[1]^ (2016)^[31]^	2235	63.0	71.0	Highest quartile vs lowest	Patients with stable coronary artery disease	Up to 5 yr	3.8; 2.5–6.5 (IQR)	All-cause mortality
Shafi, (2017)^[32]^	1232 (whites: 431; blacks: 801)	57.7	43.3	Highest quintile vs lowest	Hemodialysis patients	2.3 yr	101.9 (whites: 98.4; blacks: 103.8); 62 to 124 (whites: 63–120; blacks: 62–125) (IQR); (whites: 6.42–468; blacks: 2.25–682) (ranges)	Cardiac death, all-cause mortality
Robinson-Cohen, (2016)^[33]^	339	57.3	69.0	Highest tertile vs lowest	Patients with CKD	3.3 yr (median)	23.5; >0–> 133 (ranges)	All-cause mortality
Ottiger, (2016)^[34]^	317	72.0	59.7	Highest quartile vs lowest	Community-acquired pneumonia patients	5 yr	3.0; 1.7–5.4 (IQR)	All-cause mortality
Senthong,^[2]^ (2016)^[35]^	821	66	66	Highest quartile vs lowest	Patients with peripheral artery disease	5 yr	4.8; 2.9–8 (IQR)	All-cause mortality
Tang,^[4]^ (2017)^[36]^	1216	64.4	58	Highest tertile vs lowest	Patients with type 2 diabetes mellitus who underwent elective diagnostic coronary angiography	3 yr	4.4; 2.8–7.7 (IQR)	All-cause mortality, MACE (death, nonfatal MI, and nonfatal stroke)
Li,^[1]^ (2017)^[37]^	530	62.4	57.5	Highest quartile vs lowest	Patients with acute chest pain	1 yr	4.28; 2.55–7.91 (IQR)	All-cause mortality
	1683	63.9	77.8	Highest quartile vs lowest	Patients who underwent coronary angiography for acute coronary syndrome	NR	2.87; 1.94–4.85 (IQR)	MACE (myocardial infarction, stroke, need for revascularization, or death)
Guasch-Ferre, (2017)^[38]^	980	68.2	46.1	Highest quartile vs lowest	55 to 80 years and at high cardiovascular risk but free of CVD at baseline	4.8 yr	NR	Cardiovascular events (myocardial infarction, stroke, or death from cardiovascular causes)
Zho, (2020)^[39]^	1208	73	68.5	Highest quartile vs lowest	Patients with congestive heart failure after MI	672 d	4.5	MACE (all-cause mortality, HF rehospitalization, or recurrent MI), all-cause mortality
Croyal, (2020)^[40]^	1463	65	58%	Highest quartile vs remaining quartiles	Patients with type 2 diabetes mellitus	85 months	NR	MACE (composite of CV death, nonfatal MI, nonfatal stroke), all-cause mortality
Lee, (2021)^[41]^	4131	72.2	36	Highest quintile vs lowest	Participants free of prevalent cardiovascular disease	15 yr	4.72; 3.19–7.69 (IQR)	ASCVD (myocardial infarction, fatal coronary heart disease, stroke, sudden cardiac death, or other atherosclerotic death)
	1449	73.6	61.8	Highest quintile vs lowest	Participants with prevalent cardiovascular disease	NR	5.43; 3.57–8.74 (IQR)	Same as above
Li,^[2]^ (2022)^[42]^	1203	61.1	80.0	Highest tertile vs lowest	Patients with Acute MI	739 d	6.6; 4.0–11.6 (IQR)	MACE (composite of all-cause death, recurrence of MI, rehospitalization caused by HF, ischemic stroke, and any revascularization), all-cause mortality
Sanchez-gimenez, (2022)^[43]^	309	64.9	71.2	Highest tertile vs lowest	Patients with Acute coronary syndrome	6.7 yr	9.98; 7.42–19.19 (IQR)	MACE (myocardial infarction, hospitalization for heart failure, and all-cause mortality)
Fretts, (2022)^[44]^	5333	73	40.3	Highest quintile vs lowest	Adults aged 65 years or older	13.2 yr	4.86; 0.01–255.00 (IQR)	CVD death, all-cause mortality
Wei, (2022)^[45]^	915	57.1	69.9	Highest tertile vs lowest	Patients with chronic HF with reduced ejection fraction (HFrEF)	33 months	2.52; 1.20–4.76 (IQR)	Composite outcome of cardiovascular death or heart transplantation
Li,^[3]^ (2022)^[46]^	985	63.0	77.8	Highest tertile vs lowest	Patients with acute MI complicated by HF	716 d	6.7; 4.0–11.7 (IQR)	MACE (including all-cause death, recurrence of myocardial infarction, and rehospitalization due to HF), all-cause mortality
Chang, (2022)^[47]^	513	NR	NR	Highest quartile vs lowest	Peritoneal dialysis patients	NR	72.3; 43.7–124.7 (IQR)	CVD mortality, all-cause mortality
Luciani, (2023)^[48]^	2379	73.3	73	Highest tertile vs lowest	Previously documented AF in patients of age > 65	4 yr	NR	CVD mortality, all-cause mortality

ASCVD = atherosclerotic cardiovascular disease, CV = cardiovascular, CVD = cardiovascular disease, HF = heart failure, MACE = major adverse cardiovascular events, MI = myocardial infarction.

### 3.2. TMAO levels with the risk of MACE and all-cause mortality

**Figure 2.**
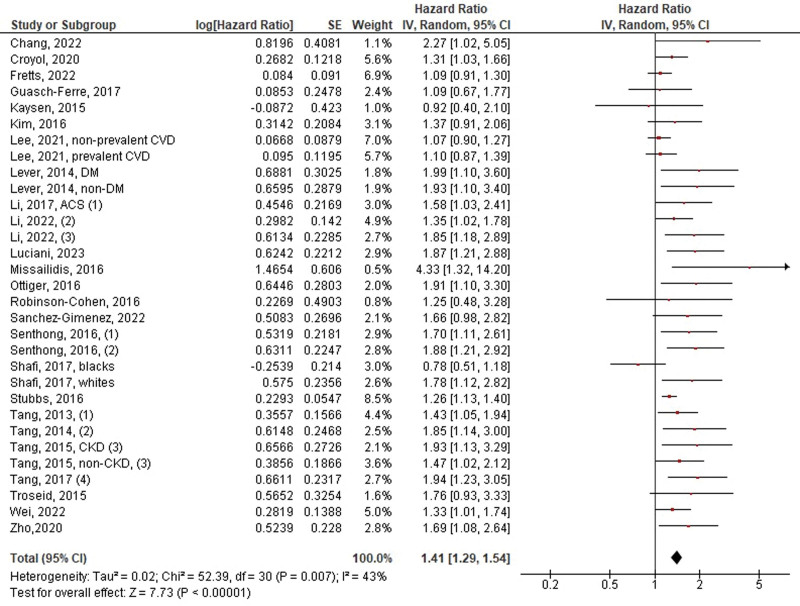
Pooled hazard ratio of TMAO levels on the risk of MACE/all-cause death. MACE = major adverse cardiovascular events, TMAO = trimethylamine N-oxide.

**Figure 3.**
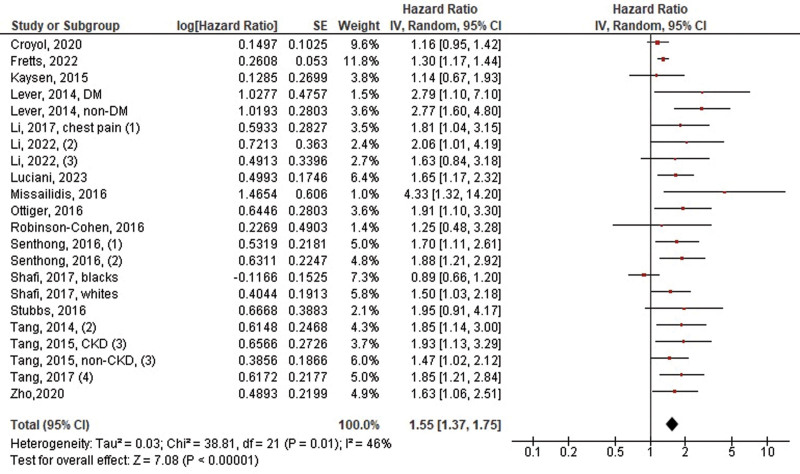
Pooled hazard ratios of TMAO levels on the risk of all-cause death alone. TMAO = trimethylamine N-oxide.

A total of 27 studies with 31 data points reported on the outcome of MACE. Of the 27 studies, 26 reported multivariate HRs, whereas only one reported crude HR. Elevated TMAO levels were associated with a significantly greater risk of MACE compared to low TMAO levels (HR: 1.41, 95% CI 1.29–1.54, *P* < .00001, *I*^2^ = 43%) (Fig. 2). Regarding all-cause mortality alone, 19 of the 27 studies reported it as an outcome. A significant risk reduction in all-cause mortality was observed with low TMAO levels compared with high TMAO levels (HR: 1.55, 95% CI 1.37–1.75, *P* < .00001, *I*^2^ = 46%) (Fig. 3).

### 3.3. Association of TMAO precursors with the risk of MACE

**Figure 4.**
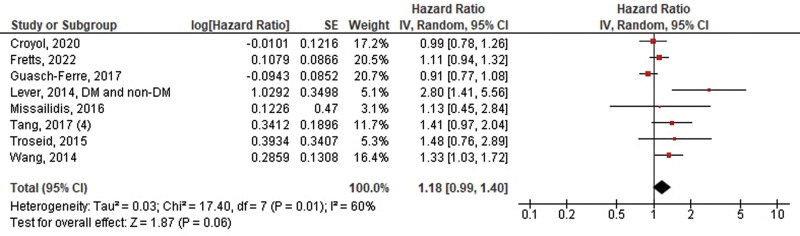
Pooled hazard ratios of betaine levels on the risk of MACE/all-cause death. MACE = major adverse cardiovascular events.

**Figure 5.**
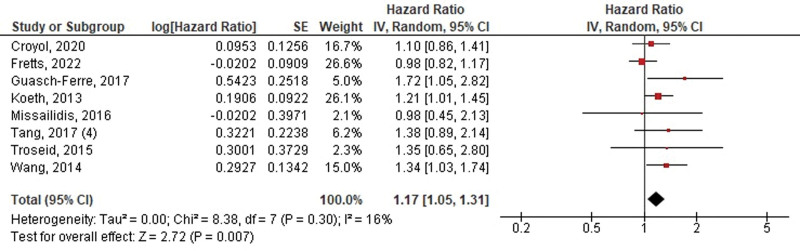
Pooled hazard ratios of choline/L-carnitine levels on the risk of MACE/all-cause death. MACE = major adverse cardiovascular events.

A total of 8 studies evaluated the risk of MACE in association with betaine and L-carnitine or choline. No significant difference was observed between high and low levels of betaine for the outcome of MACE (HR: 1.18, 95% CI 0.99–1.40, *P* = .06, *I*^2^ = 60%) (Fig. 4). However, our overall analysis demonstrated a significant increase in the risk of MACE for high levels of either L-carnitine or choline (HR: 1.17, 95% CI 1.05–1.31, *P* = .007, *I*^2^ = 16%) (Fig. 5).

### 3.4. 1 SD Log-Transformed Increment of TMAO for MACE

**Figure 6.**
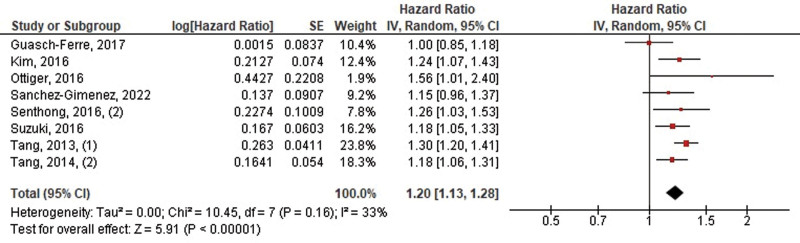
Pooled hazard ratios of 1 SD log-transformed increment of TMAO for MACE (adjusted). MACE = major adverse cardiovascular events, TMAO = trimethylamine N-oxide.

**Figure 7.**
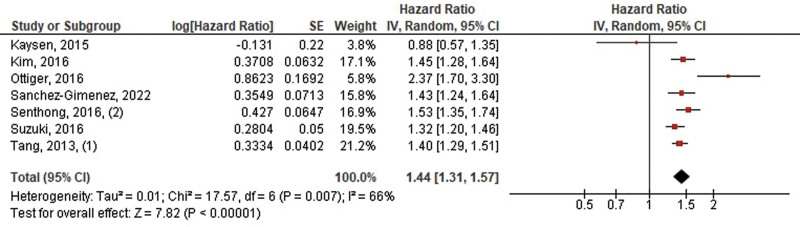
Pooled hazard ratios of 1 SD log-transformed increment of TMAO for MACE (unadjusted). MACE = major adverse cardiovascular events, TMAO = trimethylamine N-oxide.

The pooled HRs per 1 SD log-transformed increment of TMAO for MACE from 8 studies was 1.20 (95% CI, 1.13–1.28, *P* < .00001, *I*^2^ = 33%) for the adjusted model and 1.44 (95% CI, 1.31–1.57, *P* < .00001, *I*^2^ = 66%) for the unadjusted model (Figs. 6 and 7).

### 3.5. Sensitivity analysis

A sensitivity analysis assessed TMAO levels with the risk of MACE by excluding all studies that only reported data on all-cause mortality. High TMAO levels were found to be significantly associated with the risk of MACE compared to low TMAO levels after the exclusion of these studies (HR: 1.36, 95% CI 1.23–1.51, *P* < .00001, *I*^2^ = 44%) (Figure S1, Supplemental Digital Content, http://links.lww.com/MD/M230). Furthermore, the removal of outliers from the analysis of unadjusted 1 SD log-transformed increment of TMAO for MACE and association of betaine with MACE caused significant reductions in heterogeneity (*I*^2^ = 66% to *I*^2^ = 38% and *I*^2^ = 60% to *I*^2^ = 40%, respectively) (Figure S2, Supplemental Digital Content, http://links.lww.com/MD/M231 and Figure S3, Supplemental Digital Content, http://links.lww.com/MD/M232).

## 4. Discussion

The principal findings of this meta-analysis of 30 prospective studies demonstrate that elevated levels of TMAO are significantly associated with an increased risk of MACE and all-cause mortality compared with low levels. Similarly, our findings report a significant increase in the risk of MACE with high TMAO precursors, L-carnitine or choline, compared to low levels. However, no significant difference was established between high and low levels of betaine on the risk of MACE. Furthermore, the results of our analysis demonstrate a significant increase in MACE per 1 SD log-transformed increment of TMAO in both the adjusted and unadjusted models.

The relationship between blood concentrations of TMAO with MACE and all-cause mortality, established by our analysis, is in concordance with the findings of a previous meta-analysis conducted by Heianza et al^[15]^ Compared to low circulating levels of TMAO, elevated levels were associated with a 62% increased risk for the development of MACE and a 63% risk for the outcome of all-cause mortality. Our analysis established a 41% and 55% increase in the risk of MACE and mortality, respectively, for high TMAO levels. These comparable findings further strengthen the correspondence between TMAO concentrations and the development of cardiac events. Furthermore, similar to our analysis, Heianza et al^[15]^ reported a dose-dependent association between the primary outcome of MACE and TMAO levels. It is essential to mention that although higher circulating levels of the precursor L-carnitine or choline resulted in an increased risk of MACE in both studies, our analysis did not find a statistically significant difference in the risk of MACE based on betaine levels. This contrasts the findings of Heianza et al,^[15]^ which evaluated a 1.4-fold higher risk of MACE with elevated betaine levels.

There is evidence to support the risk of establishment of cardiovascular diseases with elevated TMAO levels. It is important to highlight potential explanations for this increasingly documented relationship. A study by Zeng et al^[49]^ documented a total of 17,829 all-cause and 4359 cardiovascular diseases deaths and found that a higher phosphatidyl-choline intake was associated with an increased risk of mortality (*P*-trend across quintiles < 0.0001 each). Phosphatidyl-choline is a primary dietary source of the gut microbiota-derived metabolite TMAO.^[50]^ Furthermore, several studies elucidate a pro-atherosclerotic effect of plasma TMAO.^[51]^ It is suggested that TMAO increases the cell surface expression of proatherogenic scavenger receptors,^[52,53]^ induces endothelial inflammatory injury,^[54–56]^ and augments platelet reactivity and thrombosis.^[57]^ Moreover, TMAO also has been suggested to play a role in sterol and cholesterol metabolism.^[58]^ Xiong et al^[59]^ found a significantly positive correlation between TMAO and triglycerides (*P* < .05) and a negative association between TMAO and high-density lipoprotein cholesterol (HDL-c). These processes may impact the development of cardiovascular diseases and increase the incidence of atherosclerotic cardiac events in patients with high levels of TMAO, as shown by Senthong et al.^[60]^

Regarding TMAO precursors, our investigation demonstrates a significant increase in the risk of MACE for elevated levels of choline/L-carnitine. However, a significant difference was observed based on betaine levels. A sensitivity analysis was performed by excluding an outlier study, Lever et al,^[20]^ although this did not produce a statistically significant result. Both betaine and L-carnitine are transformed to TMAO by their respective processes. The administration of dietary betaine induces the production of TMAO in animals, as suggested by Wang et al^[22]^ L-carnitine is converted to gamma-butyrobetaine by gut microbiota and subsequently to TMA and TMAO.^[50]^ It is essential to mention that only 8 out of the 30 studies enrolled in our analysis reported HRs for the risk of MACE for betaine levels. It is crucial for future investigations to establish concrete evidence for this particular association to validate the results of our study.

Our study has several strengths that need to be discussed. Firstly, including prospective studies minimizes the bias that may occur with the reverse causation phenomenon. Secondly, we performed a sensitivity analysis for the outcome of MACE by removing all studies that reported all-cause mortality to ascertain only the relationship between elevated TMAO levels and cardiac-specific events. Thirdly, unlike previous studies, we have also incorporated data from the general population. However, the sample size of this investigation still needs to be higher.

While our study adds evidence to the existing literature, some limitations must be addressed. Since all the studies included in our analysis were observational, the possibility of residual confounding could not be excluded completely. Furthermore, blood levels of TMAO may be influenced by certain environmental factors such as dietary intake.^[50,61]^ It is essential for future investigations to assess the influence of dietary intake on TMAO levels and, subsequently, the risk of MACE. Moreover, the risk of MACE with elevated TMAO levels may vary according to race, which our analysis could not account for. Lastly, since TMAO levels were measured during a single time point, the enduring long-term concentrations of these metabolites could not be accounted for.

## 5. Conclusion

Our meta-analysis implies that elevated circulating levels of gut microbiota metabolites such as TMAO and its precursor, choline or L-carnitine, are associated with an increased risk of MACE and all-cause mortality. However, no significant difference was obtained when comparing high and low levels of betaine for the risk of MACE.

## Author contributions

**Conceptualization:** Qaisar Ali Khan, Muhammad Asad.

**Data curation:** Qaisar Ali Khan, Abdul Hannan Ali, Usama Naseem, Bader Semakieh.

**Writing – original draft:** Ameer Mustafa Farrukh, Yaxel Levin-Carrion, Muhammad Afzal.

**Writing – review & editing:** Qaisar Ali Khan, Muhammad Asad.

## Supplementary Material














