# Supplementary material for: Gut microbiota metabolites and risk of major adverse cardiovascular events and death: A systematic review and meta-analysis
Source: Medicine (Baltimore). 2024 May 31;103(22):e37825. doi: 10.1097/MD.0000000000037825 (PMC11142832; doi:10.1097/MD.0000000000037825)
Supplement: Supplementary file 1 [file medi-103-e37825-s001.docx]

**Table S1. Detailed literature search of each database**

| **PubMed inception-2023, full texts** | **trimethylamine n‐oxide [text] OR TMAO [text]) AND (atherosclerosis [text] OR death [text] OR mortality [text] OR stroke [text] OR heart failure [text] OR coronary [text], cardiovascular [text] OR Cerebrovascular Disorders [Mesh] OR Cardiovascular Diseases [Mesh]** |
| --- | --- |
| **Scopus inception-2023** | **(trimethylamine n‐oxide OR TMAO) AND (atherosclerosis OR death OR mortality OR stroke OR heart failure OR coronary OR cardiovascular OR Cerebrovascular Disorders OR Cardiovascular Diseases)** |
| **Cochrane** | **(trimethylamine n‐oxide OR TMAO)** |
