# Supplementary material for: Gut microbiota metabolites and risk of major adverse cardiovascular events and death: A systematic review and meta-analysis
Source: Medicine (Baltimore). 2024 May 31;103(22):e37825. doi: 10.1097/MD.0000000000037825 (PMC11142832; doi:10.1097/MD.0000000000037825)
Supplement: Supplementary file 2 [file medi-103-e37825-s002.docx]

**Table S2. Quality assessment of included studies**

|  | Selection Comparability | | | | | Outcome | | | |
| --- | --- | --- | --- | --- | --- | --- | --- | --- | --- |
| Study | Representative nest of the exposed cohort | Selection of the non-exposed cohort | Ascertainment of exposure | Outcome not present at baseline | Comparability of the cohort | Assessment of outcome | Enough follow up duration | Adequate follow-up | Total score |
| Tang (1),  (2013) | * | * | * |  | * | * | * | * | 7 |
| Koeth,  (2013) |  | * | * | * | * | * | * | * | 7 |
| Lever,  (2014) | * | * | * |  | ** | * | * | * | 8 |
| Tang (2),  (2014) | * | * | * |  | ** | * | * |  | 7 |
| Wang,  (2014) | * | * | * |  | ** | * | * | * | 8 |
| Kaysen,  (2015) |  | * | * | * | * | * | * | * | 7 |
| Troseid,  (2015) | * | * | * | * | ** | * | * | * | 9 |
| Tang (3),  (2015) | * | * | * | * | * | * | * | * | 8 |
| Suzuki,  (2016) | * | * | * |  | * | * | * | * | 7 |
| Missailidis, (2016) | * | * | * | * | ** | * | * | * | 9 |
| Stubbs,  (2016) | * | * | * |  | * | * | * | * | 7 |
| Kim,  (2016) | * | * | * |  | ** | * | * | * | 8 |
| Senthong (1),  (2016) | * | * | * | * | ** | * | * | * | 9 |
| Shafi,  (2017) | * | * | * |  | * | * | * | * | 8 |
|  |  |  |  |  |  |  |  |  |  |
| Robinson‐Cohen,  (2016) | * | * | * |  | ** | * | * | * | 8 |
| Ottiger,  (2016) | * | * | * |  | ** | * | * | * | 8 |
| Senthong (2),  (2016) | * | * | * | * | ** | * | * | * | 9 |
| Tang (4),  (2017) | * | * | * | * | ** | * | * | * | 9 |
| Li (1),  (2017) | * | * | * |  | * | * | * | * | 7 |
|  |  |  |  |  |  |  |  |  |  |
| Guasch-Ferre, (2017) | * | * | * | * | * | * | * | * | 8 |
| Zho,  (2020) | * | * | * | * | * | * | * | * |  |
| Croyal,  (2020) | * | * | * | * | ** | * | * | * | 9 |
| Lee,  (2021) | * | * | * |  | ** | * | * | * | 8 |
|  |  |  |  |  |  |  |  |  |  |
| Li, (2),  (2022) | * | * | * |  | ** | * | * | * | 8 |
| Sanchez-gimenez,  (2022) | * | * | * |  | * | * | * | * | 7 |
| Fretts,  (2022) | * | * | * | * | ** | * | * | * | 9 |
| Wei,  (2022) | * | * | * | * | ** | * | * | * | 9 |
| Li, (3),  (2022) | * | * | * |  | ** | * | * | * | 8 |
| Chang,  (2022) | * | * | * |  | ** | * | * | * | 8 |
| Luciani,  (2023) | * | * | * | * | ** | * | * | * | 9 |
