# Supplementary figures and images for: Gut microbiota metabolites and risk of major adverse cardiovascular events and death: A systematic review and meta-analysis
Source: Medicine (Baltimore). 2024 May 31;103(22):e37825. doi: 10.1097/MD.0000000000037825 (PMC11142832; doi:10.1097/MD.0000000000037825)

**
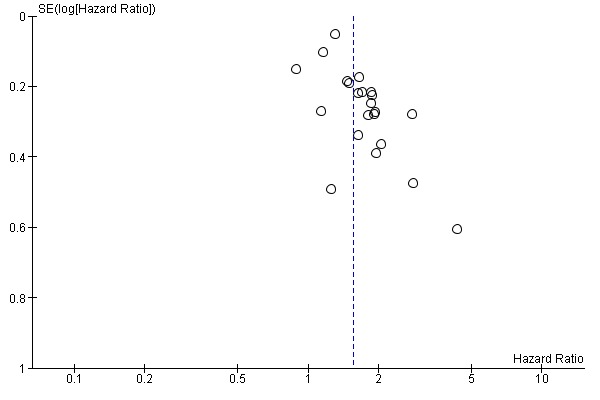
**

Figure. S4

Supplement: Supplementary file 3 [file medi-103-e37825-s003.docx]

**
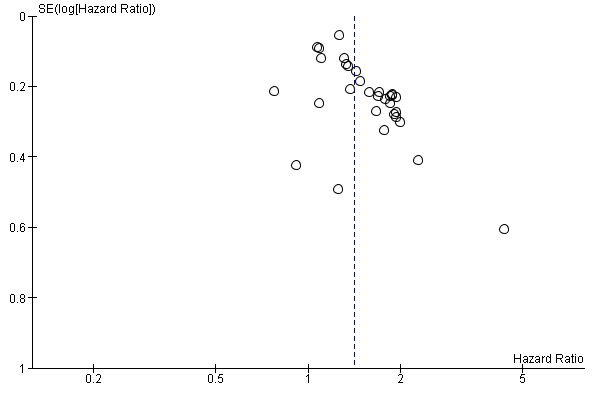
**

Figure: S5

Supplement: Supplementary file 4 [file medi-103-e37825-s004.docx]

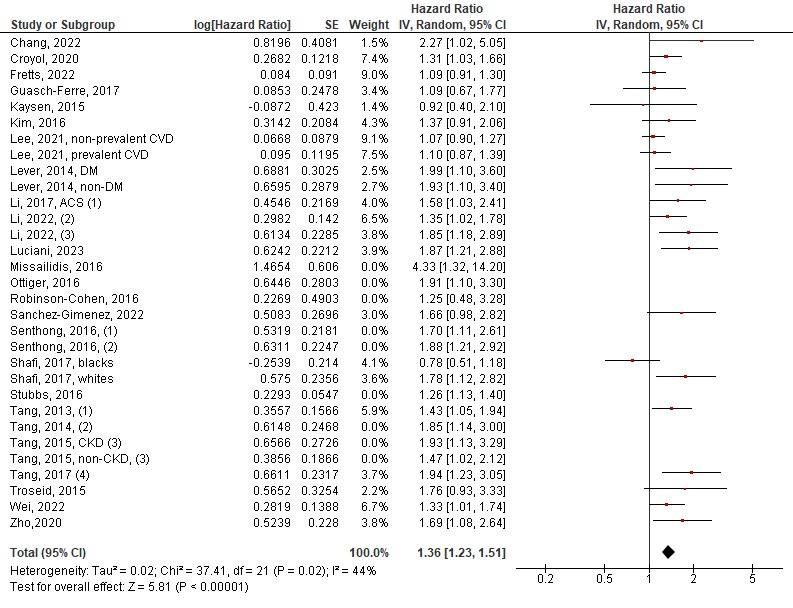


Figure. S1:

Supplement: Supplementary file 5 [file medi-103-e37825-s005.docx]

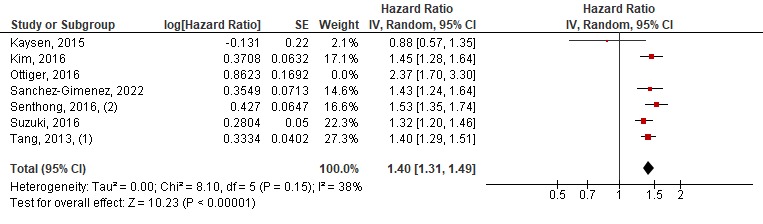


Figure: S2ß

Supplement: Supplementary file 6 [file medi-103-e37825-s006.docx]

**
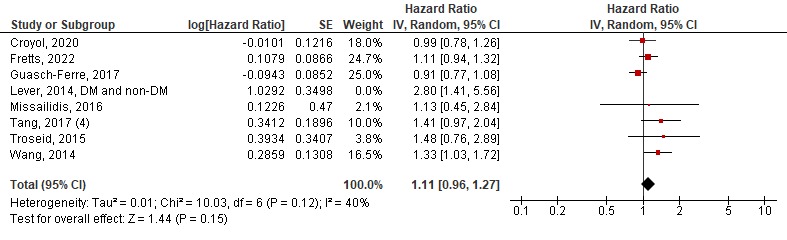
**

Figure: S3

Supplement: Supplementary file 7 [file medi-103-e37825-s007.docx]
